# OPTIMIZING PHYSICAL FITNESS IN CHRONIC STROKE PATIENTS: THE IMPACT OF EXERCISE TRAINING MODALITY AND DOSAGE ON MAXIMAL AND SUB-MAXIMAL FITNESS – A SYSTEMATIC REVIEW AND META-ANALYSIS

**DOI:** 10.2340/jrm.v57.43359

**Published:** 2025-08-11

**Authors:** Felix NINDORERA, Clement LEVEQUE, Eric MEYER, Balestra COSTANTINO, Sigrid THEUNISSEN

**Affiliations:** 1 Environmental, Occupational, Aging (Integrative) Physiology Laboratory, Department of Physiotherapy, Haute Ecole Bruxelles-Brabant (HE2B), Brussels, Belgium; 2 Centre for Interdisciplinary Research in Rehabilitation and Social Integration, Centre intégré universitaire de Santé et de services sociaux de la Capitale-Nationale, Québec, Canada; 3 Motor Sciences Department, Physical Activity Teaching Unit, Université Libre de Bruxelles (ULB), Brussels, Belgium

**Keywords:** Chronic Stroke, exercise intensity, fitness, exercise modalities

## Abstract

**Objective:**

To evaluate the effects of different exercise training modalities on maximal and sub-maximal physical fitness in chronic stroke patients and determine the optimal training dosage.

**Design:**

Systematic review and meta-analysis of 38 randomized controlled trials.

**Methods:**

A comprehensive search was conducted across seven databases (MedLine, Embase, ScienceDirect, Cochrane Library, CINAHL, and SPORTSDiscus) up to March 31, 2024. Maximal fitness was measured by VO2 max/peak, and sub-maximal fitness by the 6- or 12-minute walk test (6MWT)

**Results:**

Aerobic and mixed training significantly improved VO2 max/peak (MD = 3.16 [2.83, 3.49], *p* < 0.00001; I² = 22%). Only aerobic training significantly enhanced 6MWT performance (MD = 34.30 [25.08, 43.53], *p* < 0.00001; I² = 25%). Sensitivity analysis revealed that VO2 max/peak gains were greater with moderate-to-high intensity, while moderate intensity sufficed for 6MWT improvement. The optimal regimen was 45-minute sessions of moderate-to-high intensity aerobic training, at least three times weekly for a minimum of eight weeks.

**Conclusion:**

Moderate-to-vigorous aerobic training enhances physical fitness in chronic stroke. High-intensity and mixed training programs yield greater maximal fitness improvements, while moderate intensity benefits sub-maximal capacity. Targeted, intensity-monitored exercise programs of ≥45 minutes, three times weekly over ≥8 weeks, are recommended for significant fitness gains.

## LAY ABSTRACT

Stroke recovery presents significant challenges, particularly in regaining physical fitness. This study examined the effects of various exercise training modalities on cardiorespiratory fitness and walking ability in stroke survivors. Data from 38 randomized controlled trials were analysed to identify the most effective type and dosage of exercise for post-stroke fitness improvement. The results indicate that a combination of aerobic and resistance training (mixed training) was shown to enhance overall fitness, whereas aerobic training alone yields significant improvements in walking ability. Moderate to high-intensity aerobic exercises, such as brisk walking or cycling, performed for 45-min sessions at least 3 times per week over 8 weeks, led to significant improvements. These results underscore the value of structured and intensity-monitored exercise interventions in stroke rehabilitation. The findings may inform the development of more effective, evidence-based rehabilitation protocols for enhancing physical fitness in this population.

Stroke is a major global health burden, defined by the World Health Organization (WHO) as a “rapidly developed clinical sign of focal (or global) disturbance of cerebral function, lasting more than 24 hours or leading to death, with no apparent cause other than of vascular origin” (1, 2). With a prevalence exceeding 100 million cases worldwide and an annual incidence of over 12 million, stroke remains the second leading cause of death and the primary cause of long-term adult disability (1). Projections estimate a 27% increase in stroke incidence, prevalence, and disability-adjusted life years (DALYs) by 2047 in Europe (3).

The WHO’s International Classification of Functioning, Disability and Health (ICF) recognizes disability as a nonlinear interaction between impairments, activity limitation, and participation restrictions, influenced by a person’s health condition and contextual factors (4). Post-stroke activity limitations, particularly in the mobility domain, affect 50–82% of survivors across various areas, including basic self-care and instrumental activities of daily living (IADLs), among others (5, 6). Notably, more than 21% of individuals experience a decline in mobility within the first year post-stroke, primarily due to physical inactivity (7). Reduced mobility leads to a sedentary lifestyle, resulting in a self-perpetuating cycle of declining physical fitness and reduced ambulatory activity (8). Physical inactivity is also associated with a heightened risk of cardiovascular disease and recurrent stroke (9). Recent findings have identified fatigue, cardiorespiratory deconditioning, and reduced mobility as critical contributors to diminished physical fitness in stroke survivors (10, 11). Importantly, post-stroke fatigue, reported in 35% to 92% of individuals, further exacerbates inactivity and functional decline, reinforcing the downward spiral in physical fitness (11).

Physical fitness, defined as the capacity to perform daily tasks efficiently with vigour and alertness, without excessive fatigue, and with sufficient energy to engage in leisure activities and handle emergencies (12), is crucial for independent mobility. Cardiorespiratory fitness, as measured by maximal oxygen uptake (VO_2_ max, VO_2_ peak) is considered the gold standard for assessing physical fitness and exercise capacity, reflecting the body’s maximal rate of oxygen consumption during exertion (13). VO_2_ max has a strong inverse relationship with stroke risk (14). Stroke survivors, including individuals with only mild impairments, exhibit a reduced VO_2_ max or peak by 27–87% compared with healthy individuals of the same sex and age (15, 16). In the chronic phase of stroke (≥ 6 months), physical fitness remains reduced by 25% to 45% of the expected value (17). Engagement in regular physical activity is a key strategy to mitigate post-stroke physical deconditioning and enhance daily function (18, 19). International guidelines recommend that stroke survivors participate in 20–60 min of moderate-to-high intensity aerobic exercise (55–80% of maximal heart rate) 3–5 times per week, along with moderate-to-high intensity resistance training (50–80% of one-repetition maximum) 2–3 times per week (18, 20). Nevertheless, adherence to these guidelines remains low among stroke survivors, primarily due to barriers such as low motivation, environmental limitations, health-related concerns, and residual impairments (21). Additionally, few clinicians have experience with the assessment of cardiorespiratory fitness and exercise prescription in post-stroke populations (22).

Despite advances in understanding the importance of physical fitness and efficacy of high-intensity exercise post-stroke, few interventions are specifically designed to elicit maximal exertion, often opting for submaximal exercise to ensure patient safety (23, 24). Additionally, fitness assessments in stroke rehabilitation frequently rely on mobility tests (e.g., the 6-minute walk test, 6MWT) rather than direct measurements of VO_2_ peak, which can limit the precision of evaluating fitness-targeted interventions’ effects (25). Saunders et al. (2020) in their updated meta-analysis included fitness outcome as a secondary measure, without specifically differentiating between maximal and submaximal fitness improvements (26). Furthermore, this meta-analysis has pooled stroke patients across all stages, overlooking potential differences in training responsiveness between the subacute and chronic phases.

The present study aims to update the literature by systematically evaluating the effects of various exercise modalities on maximal and submaximal fitness in individuals with chronic stroke. It further seeks to guide clinical assessment practices based on targeted training outcomes and to identify optimal exercise dosages for improving maximal physical fitness in this population.

## METHODS

### Study design

This systematic review and meta-analysis was conducted under a pre-established protocol registered in the International Prospective Register of Systematic Reviews (PROSPERO) under registration number CRD42024526008. It adheres to the Preferred Reporting Items for Systematic Reviews and Meta-Analyses (PRISMA) guidelines (27) and follows the methodological recommendations outlined in the Cochrane Handbook for Systematic Reviews of Interventions (28).

### Search strategy

A systematic search strategy was developed using the PICO (Population, Intervention, Comparator, Outcome) framework to formulate a search equation. This was initially tested and subsequently refined in consultation with a research librarian. A literature search was conducted in 7 databases, including PubMed (Medline), Embase, ScienceDirect, CINAHL, SPORTDiscus, Cochrane Library, and Scopus, from database inception to 31 March 2024. The search strategy was customized for each database, using both keywords and Medical Subject Headings (MeSH) when relevant to the database indexing system. The complete search strategy is detailed in Table SI.

### Inclusion criteria

We included randomized controlled trials (RCTs) published in English in peer-reviewed journals, including adult participants (≥ 18 years) who had experienced a stroke and were in the chronic phase (≥ 6 months post-stroke). Eligible interventions involved any form of exercise or physical activity. The American College of Sports Medicine (ACSM) defines physical activity as any skeletal muscle movement resulting in energy expenditure, while exercise refers to structured, repetitive activities performed to improve or maintain physical fitness (29). ACSM classifies exercise into 4 primary types: *aerobic*, which improves cardiovascular endurance (e.g., walking, running, swimming); *resistance*, which enhances muscular strength and endurance (e.g., weightlifting, push-ups); *flexibility*, which increases joint range of motion (e.g., stretching, yoga); and *neuromotor*, which supports balance, coordination, and agility (e.g., tai chi, balance drills). Combined training, also known as mixed training, often incorporates aerobic and resistance components within a single program (29). Comparators included rehabilitation approaches not specifically designed to improve fitness or increase energy expenditure, such as usual care, placebo, waiting list, or sham training. RCTs that directly compared 2 types of physical activity were excluded. The outcomes assessed were the VO_2peak_/VO_2max_, indicative of maximal cardiorespiratory fitness (30), and the 6- or 12-minute walk tests (6MWT, 12MWT) (31) were used as measures of submaximal cardiorespiratory fitness. VO_2_ peak/max is the highest rate of oxygen consumption achieved during intense exercise, indicating an individual’s maximal aerobic capacity. It serves as a key measure of cardiorespiratory fitness and endurance performance (30). The 6–12 MWT is a submaximal exercise test that measures the distance an individual can walk in 6 or 12 min on a flat surface. It reflects functional aerobic capacity and cardiorespiratory endurance in daily-life activities (31).

### Study selection

Study selection was independently performed by 2 authors (FN and ST). All retrieved records were imported into the Rayyan software to facilitate the selection process. After duplicate removal, FN and ST screened the remaining studies based on titles, abstracts, and outcomes. Full texts of potentially eligible articles were reviewed to determine final inclusion. Discrepancies were resolved through discussion, and if consensus was not reached, a third author (BC) was consulted.

### Data extraction

Data from the included RCTs were extracted into a structured Excel spreadsheet (Microsoft Corp, Redmond, WA, USA) by the first author, verified by the last author, and cross-checked by a third author in the event of a discrepancy. Extracted data included study characteristics (first author, publication year, and sample size), participant characteristics (age, sex, stroke type, and time since stroke), intervention details (Frequency, Intensity, Time, and Type “FITT”), control group details (type of exercise, frequency, intensity, session duration, and intervention period), and outcome measures (VO_2_peak/max, and 6MWT). For each study, mean differences (MDs) and standard deviations (SDs) of outcomes at pre-intervention, post-intervention, or mean changes were extracted for both experimental and control groups. When numerical data were unavailable, such as when presented only in graphical form or entirely missing, the corresponding authors were contacted.

### Methodological quality and risk of bias assessment

Two authors (FN and ST) independently assessed the methodological quality of included studies using the Physiotherapy Evidence Database (PEDro) scale (32). Discrepancies in scoring were resolved through discussion, with a second author (CL) consulted when necessary. The PEDro scale consists of 10 items, classifying studies as excellent (9–10), good (6–8), fair (4–5), or poor (< 4) in quality.

Risk of bias was independently assessed by FN and ST using the Cochrane Risk of Bias Tool (33). This tool evaluates bias across 7 domains: random sequence generation, allocation concealment, performance bias, detection bias, attrition bias, reporting bias, and other sources of bias (33).

### Data synthesis, subgroup, and sensitivity analysis

Quantitative data were imported into and analysed using Review Manager (RevMan 5.3; https://www.cochrane.org/learn/courses-and-resources/software) software. Subgroup analyses were conducted by stratifying studies based on intervention type (aerobic, resistance, or mixed training) and level of training intensity. To ensure the robustness of the findings, sensitivity analyses were performed by excluding studies with poor or fair methodological quality (PEDro score < 50%; < 5/10). A minimum of 3 studies was required to conduct a meta-analysis.

### Statistical analysis

Statistical analyses were performed using Review Manager version 5.3. Effect sizes were reported as either mean differences (MDs) or standardized mean differences (SMDs) with 95% confidence intervals (CIs), depending on the consistency of measurement units across studies. The SMD/MD represents the magnitude of the intervention effect relative to observed variability. Heterogeneity among studies was assessed using the I² statistic, which quantifies the proportion of variability due to heterogeneity across studies. An I² value ≥ 50% was considered indicative of substantial heterogeneity. A fixed-effects model was used when I² ≤ 50%, whereas a random-effects model was applied when I² exceeded 50%. A p-value of < 0.05 was considered statistically significant.

## RESULTS

### Study selection

**Fig. 1:**
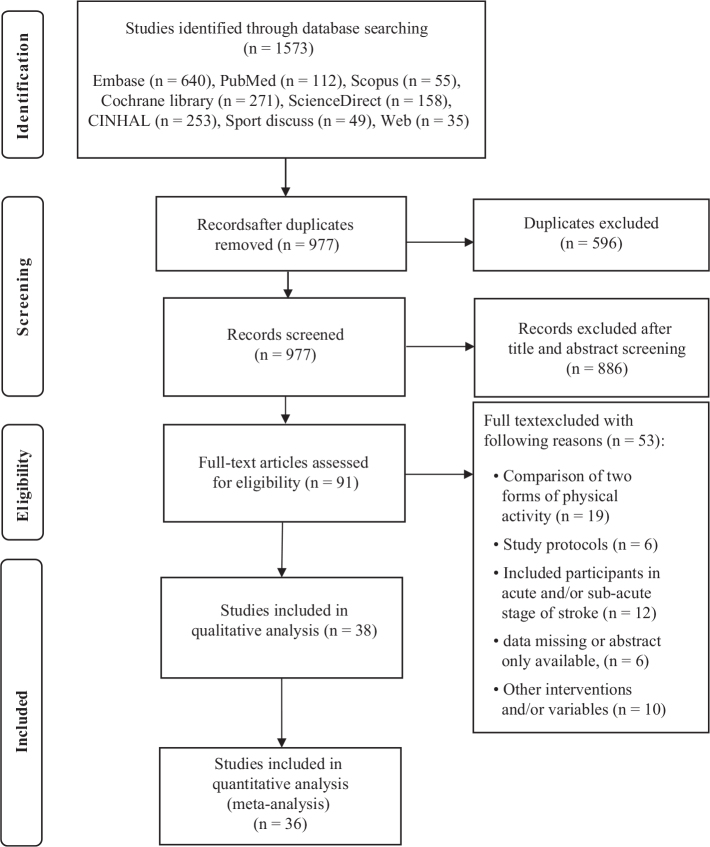
PRISMA flow diagram.

A total of 1,573 potentially relevant studies were identified through searches in the 7 electronic databases mentioned earlier. Following the removal of duplicates, titles and abstracts were screened, and full texts were reviewed. In total, 38 randomized controlled trials (RCTs) met the inclusion criteria and were included in the review (*n* = 38) and meta-analysis (*n* = 36). Two trials were excluded from the meta-analysis because essential data (means, standard deviations, or both) were not reported (34, 35). The study selection process is illustrated in Fig. 1.

The 38 included studies involved a total of 1,517 participants (709 in physical activity intervention groups and 708 in control groups). All studies assessed aerobic capacity using either a submaximal fitness test (6MWT, *n* = 25) and/or a maximal fitness test (VO_2_ peak/max, *n* = 23). The included studies were categorized into 3 groups based on their training protocols: aerobic training (*n* = 29) (34–63), resistance training (*n* = 4) (48, 55, 58, 64), and mixed training (*n* = 5) (65–69). Details of the included studies, organized chronologically from most recent to oldest, are presented in Table I. The average age of participants ranged from 44 to 75 years. Most studies were conducted in developed countries, with the following distribution: China and Taiwan (*n* = 7) (39, 46, 59–61, 64, 70), Canada (*n* = 6) (34, 40, 50, 63, 67), USA (*n* = 6) (44, 49, 53, 54, 71, 72), Australia (*n* = 5) (36–38, 66, 73), Denmark (*n* = 2) (48, 55), Germany (*n* = 2) (35, 62), and 1 study each in Norway (42), India (56), Ireland (47), and Hungary (45).

**Table I:** Outline of included studies and characteristics of each study

Study (author, year)	*n*	Intervention		Control		Duration (weeks)	Outcomes	Reported results
		Mean age (*n*)	Type	Mean age	type			
Durand UD et al. (2023)	39	54.1 ± 18.9 (*n* = 13)	Antigravity treadmill training, 45 min, 3/wk	57.9 ± 10.9 (*n* = 13)	Usual care, 45 min, 3/wk	4 weeks	Balance, 6MWT, BI, Maximum HR, VO2 max	The anti-gravity treadmill training has favourable effects on cardiorespiratory fitness
		56.1 ± 18.4 (*n* = 13)	Underwater walking therapy; 40 min, 3/wk	57.9 ± 10.9 (*n* = 13)	Usual care; 45 min, 3/wk	4 weeks	Balance, 6MWT, BI, Maximum HR, VO2 max	Non-significant changes
Lapointe et al. (2023)	52	71.8 ± 9.9 (*n* = 19)	HIIT (50% of peak power output) ergo cycle +MICT (30s at 95% peak power output & 60s passive); 4/wk; 20–40 min	69.6 ± 10.7 (*n* = 17)	Usual care, 20–40 min, 2–4/week	6 months	VO_2_ Peak, blood pressure, Hb1Ac, body composition, lipid profile (after intervention and 12 months’ follow-up)	Both HIIT+MICT and MICT only improved significantly cardiorespiratory fitness and gains were maintained in the 2 groups while control group decreased
		65.6 ± 11.3 (*n* = 16)	MICT, 4/wk, 20–40 min	69.6 ± 10.7 (*n* = 17)	Usual care			
Kang D et al. (2023)	20	54.33 ± 18.22 (*n* = 6)	Mixed exercise training (strength, cardio, game-based exercise); 3/wk, 60 min; 3 sets of 12–15 rep; 65–80% HR	56.2 ± 9.64 (*n* = 10)	No intervention 3/wk	8 weeks	6MWT, VO_2_ peak, irisin, muscle strength, and body composition	Significant improvements in leg and trunk muscle, VO_2_ peak, endurance, and body composition in the exercise group
Yeah TT et al. (2022)	56	57.36 ± 12.17 (*n* = 18)	Aerobic exercise training (stationary bicycle training); 60–70% HR; 60 min, 3/wk (and 3rd group of aerobic+cognitive training, *n* = 20)	60.1 ± 12.1 (*n* = 18)	Cognitive training, 3/wk	12 weeks	6MWT, SIS, TUG, FIM, cognitive tests, Lawton IADL	No between-group differenced were observed for physical function, daily function, quality of life, and social participation measures
Serra MC et al. (2022)	246	68 ± 2. (*n* = 20)	Aerobic treadmill rehabilitation; 60 min (at 40–60max progressive increase to 70% HRR), 3/wk	63 ± 1 (*n* = 19)	Stretching or balance training; 30 min, 2/wk	6 months	VO2 peak, 6MWT, glucose, oxidative stress, and inflammatory biomarkers	Physical function (6MWT, VO2 peak) improved after treadmill rehabilitation
Brauer SG et al. (2022)	119	62 ± 11 (*n* = 60)	Usual training+treadmill training and self-management, 30min, 40–60% HRR; 5/week	64 ± 9 (*n* = 59)	Usual gait training; 30 min, 5/week	8 weeks	Number of steps, walking ability, fitness self-efficacy	Treadmill training increased significantly steps/day but no differences between groups for other outcomes
Horvath J et al. (2022)	35	N = 10	Aerobic bicyle training (30min, 5/wk at 40-60 %HRR) + usual care (30min)	*n* = 16	Conventional rehab (occupational+physiotherapy)	4 weeks	VO_2_ peak, OUES, VO_2_-VT, 6MWT, FIM	Only submaximal outcomes were significantly improved in the intervention group
Reynolds et al. (2021)	20	57.5 ± 11.2 (*n* = 10)	Moderate intensity training (stationary bike, treadmill, stepper, recumbent bike, …) 40–59% HRR; 30 min, 2/wk	54.6 ± 8.9 (*n* = 10)	Standard care (low intensity) 10–30 min, 3/wk	12 weeks	VO_2_ peak, 6MWT, quality of life (SF-36), mood patient (Health Questionnaire, PHQ9)	Despite the improvements in both groups, no between-group differences were observed
Gjellesvik TI et al. (2021)	70	57.6 ± 9.2 (*n* = 36)	Treadmill HIIT (4x4 min at 85–95% peak HR & 4x4 min, active rest 3x4 min; at 50–70% peak HRR); 38min	58.7 ± 9.2 (*n* = 34)	Standard care, 3/week	8 weeks	VO_2_ peak; blood pressure and blood profile	24 sessions of treadmill HITT were superior to standard care in improving VO_2_ peak immediately but not at follow-up
Lapointe T et al. (2021) *(not all data are accessible)*	52	71.8 ± 9.9 (*n* = 19)	HIIT (50% of peak power output); 4/wk; 20–40min		Usual care, 20–40min, 2–4/week	6 months	VO_2_ peak, blood pressure, Hb1Ac, body composition, lipid profile (after intervention and 12 months’ follow-up)	Both HIIT and MICT provide similar significant improvements. No significant increase was noted in the control group
			MICT (30 s at 95% peak power output & 60 s passive); 4/wk; 20–40 min		Usual care, 20–40 min, 2–4/week			
Chang KW et al. (2021)	16	54.38 ± 14.5 (*n* = 8)	Usual care (30 min)+backward treadmill training (low to moderate, RPE: 0–3), 30 min, 3/week	52.39 ± 6 (*n* = 8)	Usual care, 30 min; 3/week	4 weeks	Balance (Berg balance scale), mobility (timed up and go, 10MWT, 6MWT) and pulmonary function	Experimental group showed significant differences on both measured outcomes
Linder SM et al. (2020)	44	51 ± 12 (*n* = 16)	Forced aerobic exercise and upper extremity repetitive task practice; 90 min, 3/week	58 ± 11 (*n* = 13)	Nonaerobic control group (education) 90 min, 3/week	8 weeks	VO_2_ peak	Voluntary aerobic exercise increased significantly cardiopulmonary fitness
		60 ± 14 (*n* = 15)	Voluntary aerobic exercise+upper extremity repetitive task practice 90 min, 3/week	58 ± 11 (*n* = 13)	Nonaerobic control group (education) 90 min, 3/week		VO_2_ peak	
Martins JM et al. (2020)	36	56 ± 17 (*n* = 18)	Circuit training (mixed exercises); 60 min; 3/week	55 ± 13 (*n* = 22)	Stretching+education and memory exercise	12 weeks	Walking distance (6MWT); physical activity level, human activity profile, walking speed	No significant change between groups were found
Serra MC et al. (2019)	25	58.1 ± 1.2 (*n* = 17)	Treadmill exercise, 50 min, 3/week	61.5 ± 1.3 (*n* = 8)	Whole-body stretching, 50 min, 2/wek	6 months	VO_2_ peak	Aerobic capacity was improved significantly after treadmill training
Hsu CC et al. (2019)	30	55.7 ± 3.0 (*n* = 15)	Exercise training on a bicycle ergometer, 50–60% VO_2_ peak; 30–45 min/session, 5/wk	57.8 ± 3.9 (*n* = 15)	Traditional rehabilitation, 30–45 min, 5/wk	4 weeks	VO_2_ peak, platelet, oxidative phosphorylation, electron transport chain	VO_2_ peak was increased significantly in the exercise training group
Hornby TG et al. (2019)	90	59 (*n* = 28)	High-intensity stepping training with variable stepping, 70–80% HRR; 30–40 min, 3–5/week (30 sessions)	56 (*n* = 32)	Low-intensity stepping, 30–40% HRR, 3–5/wk	2 months	6MWT, velocity, limb stance, step length	Walking parameters were significantly greater in high-intensity groups compared with low-intensity
		60 (*n* = 30)	High-intensity forward stepping walking, 70–80% HRR; 3–5/week (30 sessions)	56 (*n* = 32)	Low–intensity stepping, 30–40% HRR, 3–5/wk	2 months	6MWT, velocity, limb stance, step length	Walking parameters were significantly greater in high-intensity groups compared with low-intensity
Lund C et al. (2018)	48	67.7 ± 9.4 (*n* = 14)	Aerobic training with a cycle ergometer, IT 3x12 min, 5x10 min rest 3/week; 3/week	66.4 ± 8.8 (*n* = 16)	Sham training on upper extremity	12 weeks	Balance, gait speed, VO_2_ peak, walking endurance (6MWT)	Aerobic and resistance training improved VO_2_ peak significantly
		67.3 ± 7. 4 (*n* = 14)	Resistance training, 80% 1RM, 3/wk; 12 wks					
Vahlberg B et al. (2016)	43	72.7 ± 5.5 (*n* = 20)	Progressive resistance training+balance training, low to high intensity (10 to > 15 repetition), 90 min, 2/wk	73.7 ± 5.4 (*n* = 23)	Usual care	3 months	Body composition (fat mass and fat-free) and walking capacity (6MWT)	Three months’ progressive resistance training was associated with improved walking capacity and reduced fat mass
	67	72.6 ± 5.5 (*n* = 34)	Progressive resistance and balance training (PRB),75 min, 10 to > 15 repetitions; 2/wk	73.7 ± 5.3 (*n* = 33)	Usual care 2–3/ week	3 months	Balance, walking capacity, quality of life, physical activity level	No differences were found for the walking capacity relative to control and intervention group
Marsden DL et al. (2016)	20	54.4 ± 22. 2 (*n* = 10)	Home and community-based exercise programme; at least 30 min/day; moderate intensity, most days of weeks	62 ± 16.8 (*n* = 10)	Usual care	12 weeks	Feasibility and safety; 6MWT, VO_2_ peak during Cycle Progressive Exercise Test	VO_2_ peak and distance walked improved more in the intervention group
Moore SA et al. (2016)	40	68 ± 8 (*n* = 20)	Progressive mixed training (aerobic, strength, balance, flexibility), 40–50% HRR & 70–80% HRR; 45–60 min, 3/wk	70 ± 11 (*n* = 20)	Home stretching programme, 45–60 min, 3/wk	19 weeks	Cardiorespiratory (VO_2_ peak) and functional performance (6MWT)	Significant improvement of VO_2_ peak and walking performance in the exercise group
Srivastava A. et al. (2016)	45	44.4 ± 12.31 (*n* = 15)	Treadmill training, 20 sessions, 30 min/day, 5/week	44.2 ± 11.7 (*n* = 15)	Conventional overground training 30 min/day, 5/wk	4 weeks	Walking speed (10MWT), walking endurance (6MWT), impairments (Scandinavian stroke scale and functional ambulation category)	Non-significant differences were observed according to all variables in the 3 groups, despite the greater improvement in bodyweight support group
		47.93 ± 9.95 (*n* = 15)	Treadmill training with bodyweight support (40%), 20 sessions, 30 min/day, 5/wk					
Moore SA et al. (2015)	40	68 ± 8 (*n* = 20)	Progressive mixed training (aerobic, strength, balance, flexibility), 40–50% HRR & 70–80% HRR; 45–60 min, 3/wk	70 ± 11 (*n* = 20)	Home stretching programme, 45–60 min, 3/wk	19 weeks	Cardiorespiratory (VO_2_ peak) and functional performance (6MWT)	Cardiorespiratory fitness and physical function was improved significantly by exercise
Wang TC et al. (2015)	51	65.4 ± 10.6 (*n* = 25)	Home-based exercise intervention mediated with caregiver, 90 min, at least 2/week	62 ± 9.5 (*n* = 26)	No intervention	12 weeks	Balance, walking speed and endurance, disability level	Mobility was improved significantly in the intervention group
Yang HC et al. (2014)	31	53.6 ± 10.3 (*n* = 16)	Usual care+cycling training with Motomed, 30 min; 5/wk	54.5 ± 8.0 (*n* = 15)	Usual care	4 wks	Motor recovery (FMA-LE), 6MWT, 10MWT	Walking endurance and LE extremity functional recovery were improved by cycling training
Tang A et al. (2014)	50	65.9 ± 6.4 (*n* = 25)	High-intensity aerobic exercise, 40–60 min; 3/wk, intensity progression from 40% to 70–80% HRR	66.9 ± 7.8 (*n* = 25)	Low-intensity nonaerobic: balance and flexibility exercise, < 40%HRR	6 months	Aerobic capacity (VO_2_ peak), haemodynamic and cardiac function	No difference was noted between groups despite the greater improvements in the aerobic exercise group
Severinsen K et al. (2014)	48	69 (*n* = 13)	Aerobic training with cycle ergometer, 15 min, 3/wk, 75% HR	66 (*n* = 18)	Low-intensity sham training (upper extremity training)	12 weeks	Walking distance (6MWT) and walking speed (10MWT)	No significant changes were found between groups over time
		68 (*n* = 14)	Resistance training with resistive machine; 3/wk, 3 series of 8 rep, 80% 1RM					
Jin H et al. (2013)	128	576 ± 6.6 (*n* = 65)	Progressive aerobic cycling training; 40 min; 5/week; 50–70% heart rate reserve	56.3 ± 6.5 (*n* = 63)	Conventional rehabilitation	12 weeks	6MWT, heart rate recovery, VO_2_ peak and balance	Cycling intervention improved significantly peak VO_2_, HRR
Ada L (2013)	102	70 ± 11 (*n* = 34)	Treadmill+overground walking training, 30 min; 3/wk	63 ± 13 (*n* = 34)	No intervention	2 months	Walking distance (6MWT), walking speed (10MWT), step length and cadence, health status, participation, self-efficacy	Treadmill and overground walking training delivered for 4 months was more effective than a 2-month programme or no training in improving walking (distance and speed) and health
		64 ± 12 (*n* = 34)	Treadmill +overground walking training, 30min; 3/week	63 ± 13 (*n* = 34)	No intervention	4 months		
Globas et al. (2012)	36	68.6 ± 6.7 (*n* = 18)	High-intensity treadmill training; 50 min, 3/wk	68.7 ± 6.1 (*n* = 18)	Usual care; 50 min, 3/wk	12 weeks	VO_2_ peak, 6MWT, gait velocity, balance, and functional strength	Treadmill training group improved significantly VO_2_ peak and walking capacity more than usual care
Quaney BM et al. (2009)	38	64.1 ± 12.3 (*n* = 19)	Aerobic exercise: progressive resistive stationary bicycle training, 70% maximal heart rate; 45min; 3/week	58.9 ± 14.6 (*n* = 19)	Stretching at home	8 weeks	VO2 max, balance, velocity, motor learning	Stroke survivors in the aerobic group improved fitness significantly after intervention but no retention at follow-up
Luft AR et al. (2008)	113	63.6 ± 10 (*n* = 37)	Task-repetitive treadmill exercise, 40 min (60% HRR); 3/wk	63.2 ± 8.7 (*n* = 34)	Stretching	6 months	Walking distance (6MWT), walking velocity (10MWT), fitness (VO_2_), brain activation (fMRI)	Intervention improved walking capacity, and fitness, and promotes plasticity
Lennon O et al (2008)	48	60.5 ± 10 (*n* = 24)	Usual care+aerobic training on cycle ergometer, 30 min (50–60% HRR), 2/wk	59 ± 10.3 (*n* = 24)	Conventional rehabilitation, 30 min, 2/week	10 weeks	Cardiac risk score, fitness (VO_2_); blood pressure, fasting lipids, anxiety and depression	VO_2_ was improved significantly more in intervention participants than in controls
Yang YR et al (2006)	48	60 ± 10.4 (*n* = 24)	Task-oriented progressive resistance strength training, 30 min, 3/wk	56.8 ± 10.2 (*n* = 24)	Any rehabilitation	4 weeks	Muscle strength, gait parameters, endurance (6MWT)	All functional outcomes were significantly improved in the experimental group
Pang YC et al (2005)	63	65. 8 ± 9.1 (*n* = 32)	Fitness and mobility exercise (FAME), 60 min; 3/week;	64. 7 ± 8.4 (*n* = 31)	Upper extremity programme	19 weeks	VO_2_ max; mobility (6MWT), muscle strength, balance body composition	Intervention group had significantly more gains in cardiorespiratory fitness, mobility and paretic leg
Macko RF et al. (2005)	61	63 ± 10 (*n* = 32)	Progressive treadmill aerobic training (TAEX), 40 min (60–70%); 3/week	64 ± 8 (*n* = 29)	Usual care of stretching exercise, 40 min, 3/week	6 months	VO_2_ peak, mobility (6MWT), impairments questionnaires	Only TAEX improved cardiovascular fitness and ambulatory performances
Kamps A & Schule K (2005)	31	63.1 ± 8.1 (*n* = 16)	Aerobic training on Motomed, 13 Borg rating scale; at least 10 min; 2/wk	65.8 ± 10.7 (*n* = 15)	Standard rehabilitation	4 months	Gait velocity, walking distance (2 and 6 MWT) and balance	No significant change in walking endurance
Chu KS et al. (2004)	12	61.9 ± 9.4 (*n* = 7)	Water-based exercise programme, 60 min, 3/wk	63.4 ± 8.4 (*n* = 6)	Upper extremity function programme, 60 min, 3/wk	8 wks	VO_2_ max, muscle strength, velocity and balance	The water-based exercise programme attained significant improvements over the control group in fitness, mobility, and muscle strength
Ada L et al. (2004)	29	66 ± 11 (*n* = 13)	Treadmill and overground training; 30 min, 3/wk	66 ± 11 (*n* = 14)	Placebo low-intensity training and home exercise, 3/wk	4 weeks	Walking speed (10MWT), walking capacity (6MWT), handicap	The 4-week treadmill and overground walking programme significantly increased walking speed, capacity, and a decrease in handicap

6MWT: 6-min walk test; VO_2_ max: peak oxygen uptake; HIIT: high-intensity interval training; IT: interval training; FMA-LE: Fugl Meyer Assessment for Lower Extremity; HRR: heart rate reserve; fMRI: functional magnetic resonance imaging; FITT: Frequency, Intensity, Time, Type; wk: week.

Table II presents the Frequency, Intensity, Time, and Type (FITT) protocol for aerobic training studies. Aerobic training interventions were conducted over a median duration of 11 weeks (range: 2 to 19 weeks), with most interventions spanning 4 to 12 weeks. The average session duration was 44.1 ± 18.3 min, with most sessions lasting 30 to 50 min. The median training frequency was 3 sessions per week, and the total training duration averaged 1,429 ± 787 min (range: 392 to 3,420 min, with a predominant range of 600 to 2,160 min). Regarding training intensity, 7 studies (25%) used low-intensity training, 13 studies (47%) applied moderate intensity, and 8 studies (28%) employed high intensity, with the majority (73%) involving low-to-moderate intensity interventions. The most used aerobic training modalities were the stationary bicycle (*n* = 15) and the treadmill (*n* = 12). Additionally, overground walking, stepping exercises, and water-based training were each used in a single study.

**Table II:** Training protocol characteristics for aerobic exercise training: Frequency, Intensity, Time, and Type (FITT) parameters

Author (year)	Type & modality	Session time (min)	Frequency, times/week	Protocol duration, weeks	Intensity	Total training time (min)
Durand UD et al. (2023)	Aerobic treadmill training	45	4	4	Low	540
	Aerobic aqua training	50	3	4	Low	600
Lapointe et al. (2023)	Aerobic cycling	40	4	15	High	2,400
	Aerobic cycling	40	4	15	Moderate	2,400
Yeah TT et al. (2022)	Aerobic cycling	60	3	12	Moderate	2,160
Serra MC et al. (2022)	Aerobic treadmill training	60	2	15	Moderate	1,800
Brauer SG et al. (2022)	Aerobic treadmill training	30	5	8	Moderate	1,200
Horvath J et al. (2022)	Aerobic cycling	30	5	4	Moderate	600
Reynolds et al. (2021)	Aerobic cycling	30	2	12	Moderate	720
Gjellesvik TI et al. (2021)	Aerobic treadmill training	48	3	8	High	1,152
Chang KW et al. (2021)	Aerobic treadmill training	30	3	4	Low	360
Linder SM et al. (2020)	Aerobic cycling	90	3	8	High	2,160
	Aerobic cycling	90	3	8	Moderate	2,160
Serra MC et al. (2019)	Aerobic treadmill training	50	3	15	Moderate	2,250
Hsu CC et al. (2019)	Aerobic cycling	45	3	4	Moderate	540
Hornby TG et al. (2019)	Aerobic stepping training	35	5	8	High	1,200
Lund C et al. (2018)	Aerobic cycling	40	3	12	High	1,440
Srivastava A et al. (2016)	Aerobic treadmill training	30	5	4	Low	600
Wang TC et al. (2015)	Aerobic (walking, Mmobility exercise)	90	2	12	Low	2,160
Yang HC et al. (2014)	Aerobic cycling	30	5	4	Moderate	600
Tang A et al. (2014)	Aerobic cycling	30–40	3	15	High	1,800
Severinsen K et al. (2014)	Aerobic cycling	15	3	12	High	540
Jin H et al. (2013)	Aerobic cycling	40	5	12	High	2,400
Ada L (2013)	Aerobic treadmill training	30	3	8	Moderate	2,160
	Aerobic (walking, mobility exercise)	30	3	16	Moderate	1,710
Globas et al. (2012)	Aerobic treadmill training	50	3	12	High	1,800
Quaney BM et al. (2009)	Aerobic cycling	45	3	8	Moderate	1,080
Luft AR et al. (2008)	Aerobic treadmill training	40	3	15	Moderate	1,800
Lennon O et al. (2008)	Aerobic cycling	30	2	10	Moderate	600
Pang YC et al. (2005)	Aerobic (walking, mobility exercise)	60	3	19	Moderate	3,420
Macko RF et al. (2005)	Aerobic treadmill training	40	3	15	Moderate	1,800
Kamps A & Schule K (2005)	Aerobic cycling	20	2	16	Moderate	640
Chu KS et al. (2004)	Aerobic aqua training	60	3	8	Low	1,440
Ada L et al. (2004)	Aerobic treadmill training	30	3	4	Low	360

Table III presents the FITT protocol for resistance and mixed training studies. Resistance training protocols were conducted twice per week over a median duration of 12 weeks. Session duration ranged from 10 to 30 min, typically consisting of 3 sets of 8 to 15 repetitions. The average total training duration was 1,116 min (range: 360 to 2,160 min). Mixed training interventions were performed 3 times per week (median frequency) over an average duration of 14 weeks. The median session duration was 54 min, with a mean total training volume of 2,448 min (range: 1,440 to 2,160 min).

**Table III:** Training protocol characteristics for resistance and mixed exercise training: Frequency, Intensity, Time, and Type (FITT) parameters

Author (year)	Type & modality	Session time, min	Frequency, times/week	Protocol duration, weeks	intensity	Total training time (min)
Lund C et al. (2018)	Resistance training	3 series x 8 rep; 10–15 min	2	12	High	360
Vahlberg B et al. (2016)	Resistance training	90 min, 10–15 rep	2	12	Moderate	2,160
Vahlberg B et al. (2016)	Resistance training	75 min, 10 rep	2	12	High	1,800
Severinsen K et al. (2014)	Resistance training	3 series, 8 rep; 25 min	3	12	High	900
Yang YR et al. (2006)	Resistance training	30 min	3	4	Low	360
Total			12	52		5,580
Kang D et al. (2023)	Mixed trainings	60	3	8	High	1,440
Martins JM et al. (2020)	Mixed trainings	60	3	12	Moderate	2,160
Marsden DL et al. (2016)	Mixed trainings	30	5	12	Moderate	1,800
Moore SA et al. (2016)	Mixed trainings (ADLs, PA)	60	3	24	High	3,420
Moore SA et al. (2015)	Mixed trainings (ADLs, PA)	60	3	19	Moderate	3,420
Total		270	17	75		12,240

### Methodological quality and risk of bias assessment

The methodological quality of the included studies was predominantly rated as good, with 27 out of 38 studies (71%) achieving a score of 6 or higher on the PEDro Scale. The mean PEDro score across all studies was 6, with scores ranging from 3 to 9. Detailed methodological quality assessments for each study are presented in Table IV. Specifically, 4 studies (11%) were rated as having excellent methodological quality, 23 studies (60.5%) as good, 7 studies (19%) as fair, and 4 studies (11%) as poor.

**Table IV:** Methodological quality assessment with PEDro scale

Author (year)	A	B	C	D	E	F	G	H	I	J	Score	Level of quality
Ada L (2013)	1	1	1	1	0	1	1	1	1	1	9	I
Reynolds (2021)	1	1	1	1	0	1	1	NC	1	1	8	I
Vahlberg (2016)	1	1	1	0	0	1	1	1	1	1	8	I
Globas (2012)	1	1	1	NC	NC	1	1	1	1	1	8	I
Durand (2023)	1	1	1	0	0	0	1	1	1	1	7	II
Lapointe (2023)	1	1	1	0	0	1	1	0	1	1	7	II
Brauer (2022)	1	1	1	0	0	1	0	1	1	1	7	II
Horvath (2022)	1	1	1	NC	0	1	1	1		1	7	II
Gjellesvik (2021)	1	1	1	0	0	1	1	NC	1	1	7	II
Hsu CC (2019)	1	1	1	0	1	NC	1	NC	1	1	7	II
Hornby (2019)	1	1	1	0	0	1		1	1	1	7	II
Moore (2016)	1	1	1	0	0	1	1	NC	1	1	7	II
Yang HC (2014)	1	1	1	0	0	1	1	NC	1	1	7	II
Tang (2014)	1	1	1	0	0	1	1	0	1	1	7	II
Severinsen (2014)	1	1	1	0	0	1	1	NC	1	1	7	II
Luft (2008)	1	1	1	0	0	1	1	NC	1	1	7	II
Lennon (2008)	1	1	NC	NC	NC	1	1	1	1	1	7	II
Yeah (2022)	1	1	1	0	0	1	1	1	1	0	6	II
Linder (2020)	1	1	1	NC	NC	NC	1	NC	1	1	6	II
Martins (2020)	1	1	0	0	0	1	0	1	1	1	6	II
Lund C (2018)	1	1	1	0	0	0	0	1	1	1	6	II
Srivastava A (2016)	1	1	1	NC	NC	1	0	NC	1	1	6	II
Moore SA (2015)	1	1	1	0	0	1	1	NC	1	NC	6	II
Wang TC (2015)	1	1	NC	0	NC	1	1	NC	1	1	6	II
Quaney BM (2009)	1	1	1	1	NC	NC	NC	1	1	NC	6	II
Yang YR (2006)	1	1	1	0	0	1	1	NC	1	0	6	II
Serra MC (2022)	1	1	1	0	0	NC	0	0	1	1	5	III
Chang KW (2021)	1	1	1	0	0	0	1	NC	1	NC	5	III
Jin H et al. (2013)	1	1	1	0	0	1	NC	0	1	0	5	III
Pang YC (2005)	1	1	NC	0	0	1	0	1	1	0	5	III
Macko RF (2005)	1	1	1	0	0	1	0	0	1	0	5	III
Ada L et al. (2004)	1	1	NC	NC	NC	1	1	0	1	0	5	III
Kamps K (2005)	1	1	1	NC	NC	NC	0	0	1	0	4	III
Kang (2023)	1	1	NC	0	0	0	NC	0	1	0	3	IV
Serra MC (2019)	1	1	1	NC	NC	0	0	0	NC	NC	3	IV
Marsden DL (2016)	0	1	1	0	1	NC	0	NC	NC	0	3	IV
Chu KS (2004)	1	0	1	NC	NC	0	1	NC	NC	0	3	IV

1 = yes; 0 = no; NC = not clear; A = random allocation; B = concealed allocation; C = groups similar at baseline; D = participant blinding; E = therapist blinding; F = assessor blinding; G = < 15% dropout; H = intention-to-treat analysis; I = between-group difference reported; J = point estimate and variability reported. Quality levels: I = score 8–10 (excellent quality); II = score 6–7 (good quality); III = score 4– 5 (fair); IV = score < 4 (poor quality).

**Fig. 2:**
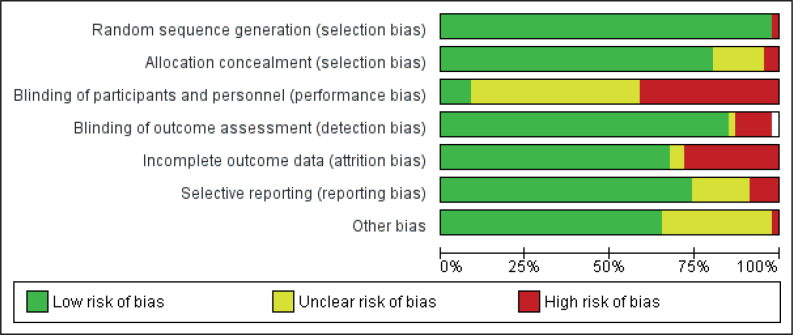
Risk of bias graph summary.

Fig. 2 and Fig. S1 detail the risk of bias assessment in the included studies. Over 75% of studies were assessed as having a low risk of bias concerning random sequence generation and allocation concealment, 83% with blinding of outcome assessment, 63.8% with incomplete outcome data, 27.7% with selective reporting, whereas 91% of studies were judged to have a high risk of bias concerning blinding of participants and personnel criteria.

### Effect of physical exercise training on maximal fitness

**Fig. 3:**
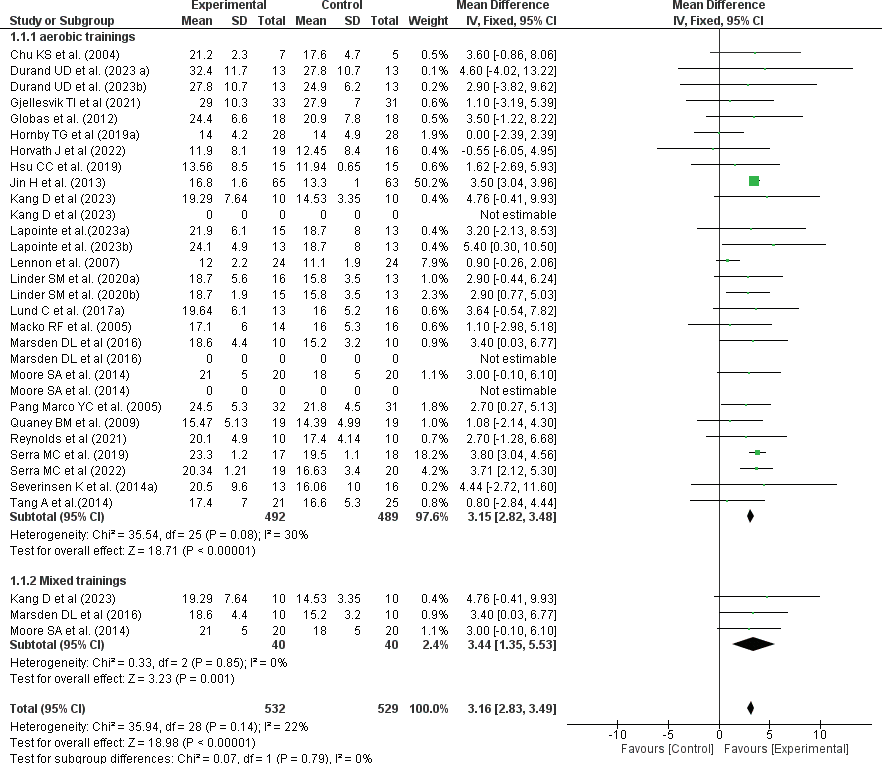
Effect of exercise on Vo_2_ max/vo_2_ peak.

Maximal fitness was assessed in 23 studies involving 1,061 stroke patients. The meta-analysis revealed a significant overall beneficial effect of interventions on VO_2_ peak/max (MD = 3.16 [2.83, 3.49], *p* < 0.00001; I² = 22%). Subgroup analysis indicated that both aerobic and mixed training significantly improved maximal fitness (*p* < 0.00001 and *p* = 0.001, respectively). Detailed effects of physical exercise training on maximal fitness are illustrated in Fig. 3.

### Effectiveness of physical exercise training on sub-maximal fitness

**Fig. 4:**
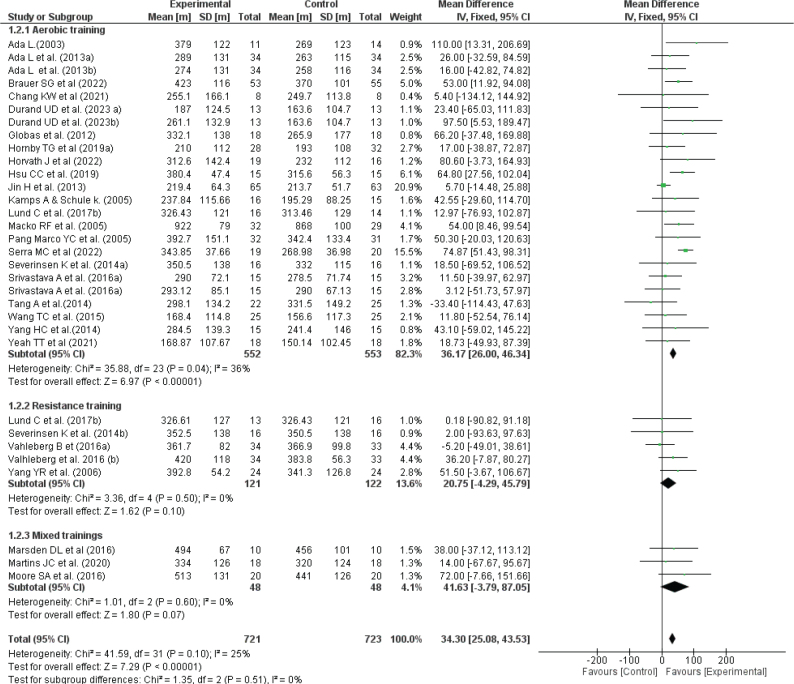
Effect of exercise on 6MWT.

Fig. 4 presents the effects of physical exercise training on submaximal fitness. Twenty-six studies evaluated walking endurance as a sub-maximal fitness outcome using the 6MWT, involving a total of 1,444 stroke patients. The meta-analysis demonstrated a significant improvement in walking distance following interventions (6MWT, MD = 34.30 [25.08, 43.53], *p* < 0.00001; I² = 25%). Subgroup analysis revealed that only aerobic training significantly enhanced sub-maximal fitness as measured by the 6MWT (MD = 36.17 [26.00, 46.34], *p* < 0.0001; I² = 36%).

### Sensitivity and meta-regression analysis

**Fig. 5:**
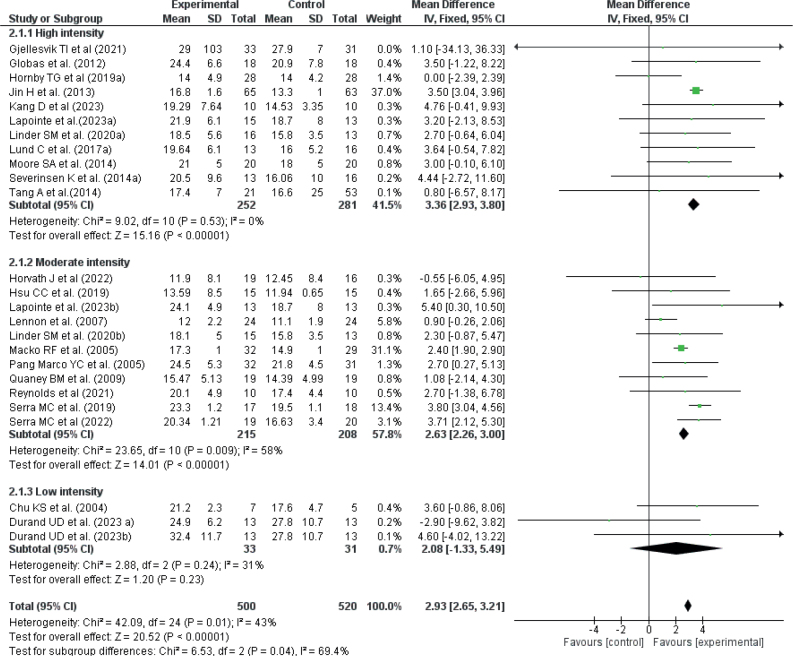
Effect of exercise intensity on VO_2_max/Vo_2_peak.

A sensitivity analysis was conducted by excluding low-quality studies and comparing studies based on intervention intensity. By removing trials with PEDro scores below 5, treatment effects remained statistically significant for both the 6MWT and VO_2_ peak/max (MD = 39.24 [10.00, 68.48], *p* < 0.00001; I² = 78% for 6MWT and MD = 1.67 [0.95, 2.39], *p* < 0.00001; I² = 0% for VO_2_ peak/max, see Fig. S2 for VO_2_ peak/max and Fig. S3 for 6MWT). Regarding intensity, high- and moderate-intensity training were most effective in improving maximal fitness (VO_2_ peak/max, MD = 2.93 [2.65, 3.21], *p* < 0.00001; I² = 43%, Fig. 5), while moderate-intensity intervention was sufficient to improve sub-maximal fitness (6MWT, MD = 34.79 [24.50, 45.08], *p* < 0.00001; I² = 40%, see Fig. S4).

A meta-regression analysis was performed with total training time (see Table II) as the dependent variable to better understand the impact of training dosage on effect size. Although no significant difference effect of total training time on effect sizes was found, interventions conducted for at least 45 min, 3 times per week, for a minimum of 8 weeks (totalling 1,080 min) demonstrated comparatively greater improvements on both the 6MWT and VO_2_ peak/max.

## DISCUSSION

This systematic review and meta-analysis sought to update existing evidence on the effectiveness of various physical exercise training protocols in chronic stroke rehabilitation and to examine the influence of training type and optimal dosage on fitness outcomes. To our knowledge, this is the first meta-analysis to compare the effects of aerobic, resistance, and mixed training on both maximal and submaximal fitness, and to examine the impact of training intensity and cumulative dose. The meta-analysis results indicated an overall improvement in maximal and submaximal fitness (VO_2_ peak/max and 6MWT) following physical activity, particularly with aerobic and mixed training. By focusing specifically on studies with good methodological quality, the findings indicate that only aerobic exercise was associated with significantly greater improvements in submaximal fitness, whereas both aerobic and mixed training were effective in enhancing maximal fitness. Additionally, both high- and moderate-aerobic or mixed training intensity showed beneficial effects on maximal capacity, whereas only aerobic moderate-intensity training was sufficiently effective for improving submaximal fitness.

### Effect of aerobic, resistance, and mixed exercise training

Findings from the current meta-analysis support the existing evidence on the effectiveness of aerobic physical training in enhancing fitness. Aerobic interventions constitute the predominant form of rehabilitation for stroke patients, in comparison with mixed or resistance training (74). Our findings are consistent with those reported in the Cochrane review by Saunders et al. (26), which indicates that moderate-intensity cardiorespiratory fitness training, particularly walking, can improve fitness, balance, and walking ability post-stroke (26). When considering studies of good methodological quality, our meta-analysis shows that resistance training does not provide additional benefits over aerobic training in improving physical fitness. This finding is corroborated by Marzolini et al., who found no significant difference in VO_2_ peak between combined aerobic and resistance training (75). Additionally, Lund et al. concluded that aerobic training remains the most effective for improving cardiorespiratory fitness, despite the minimal added value of resistance training (48). A recent network meta-analysis by Wang et al. further supports these findings that training intensity, rather than training modality, plays a more significant role in improving physical fitness, with aerobic training outperforming resistance training in this regard (76).

### Importance of intensity in stroke rehabilitation

This meta-analysis found no significant effect of low-intensity training on fitness. Moderate-intensity training resulted in statistically significant improvements in sub-maximal fitness, while high-intensity training was more effective in enhancing maximal fitness. These findings suggest that high-intensity training is effective in eliciting clinically meaningful improvements in fitness for chronic stroke patients. This is further supported by Abbasian and Rastegar (77), who demonstrated that high weekly volume of treadmill exercise training was significantly associated with greater improvement in VO_2_ peak (77). However, Lee and Stone (78) found no significant difference between moderate-intensity (40–60% of heart rate reserve [HRR]) and high-intensity (60–85% of HRR) training effects (78). Nonetheless, high-intensity training continues to show potential. A recent study involving patients more than 2 years post-stroke showed that vigorous walking exercise led to significant improvements in walking capacity after only 4 weeks of training, although at least 12 weeks of training was required to achieve greater improvements (79). Nevertheless, adherence to high-intensity training remains a significant challenge for many individuals post-stroke, potentially contributing to the limited number of high-quality studies on high-intensity protocols (73).

### Optimal dose needed to improve fitness

Our meta-analysis suggests that a 45-min training session at moderate to high intensity, performed at least 3 times per week for a minimum of 8 weeks, was associated with improvements in physical fitness. Furthermore, walking distance improved as the training period extended. Ammann et al. (80) noted that many published protocols exhibit inconsistencies and incomplete reporting of exercise training principles, as well as variations in patient adherence (80). The optimal exercise dose is influenced by multiple factors, including training intensity (high-intensity training yields short-term effects, such as those observed within 4 weeks) (79), the training modality (aerobic vs resistance training), and patient-specific characteristics (e.g., age, stroke severity, etc.) (81).

### Study limitations and strengths

Several limitations of this study should be considered. We focused only on the immediate effects of interventions, excluding follow-up data. This limitation may reduce the generalizability of our findings regarding long-term fitness outcomes. Furthermore, inconsistencies in the implementation of the FITT principle across rehabilitation protocols, in addition to high heterogeneity of the included studies, may have affected the internal validity of the findings. Finally, selection bias from inclusion/exclusion criteria and missing data may limit the generalizability of conclusions due to the omission of contextual variables such as training type and participant motivation.

### Conclusion

In conclusion, this systematic review with meta-analysis provides an update on the effectiveness of aerobic interventions in improving physical fitness. The findings suggest that moderate-to-vigorous-intensity aerobic training is most effective for enhancing overall physical fitness. High-intensity aerobic and mixed interventions demonstrated greater efficacy in improving maximal physical fitness, whereas moderate-intensity aerobic training is sufficient to enhance submaximal physical fitness. A training program consisting of 45-min sessions, at least t3 times per week, for a minimum of 8 weeks was associated with significant improvements in fitness.

## Supplementary Material




