# Supplementary material for: OPTIMIZING PHYSICAL FITNESS IN CHRONIC STROKE PATIENTS: THE IMPACT OF EXERCISE TRAINING MODALITY AND DOSAGE ON MAXIMAL AND SUB-MAXIMAL FITNESS – A SYSTEMATIC REVIEW AND META-ANALYSIS
Source: J Rehabil Med. 2025 Aug 11;57:43359. doi: 10.2340/jrm.v57.43359 (PMC12359817; doi:10.2340/jrm.v57.43359)

Supplementary material has been published as submitted. It has not been copyedited, or typeset by Journal of Rehabilitation Medicine

Fig. S1. Risk of bias summary according to Cochrane risk of bias assessment tools

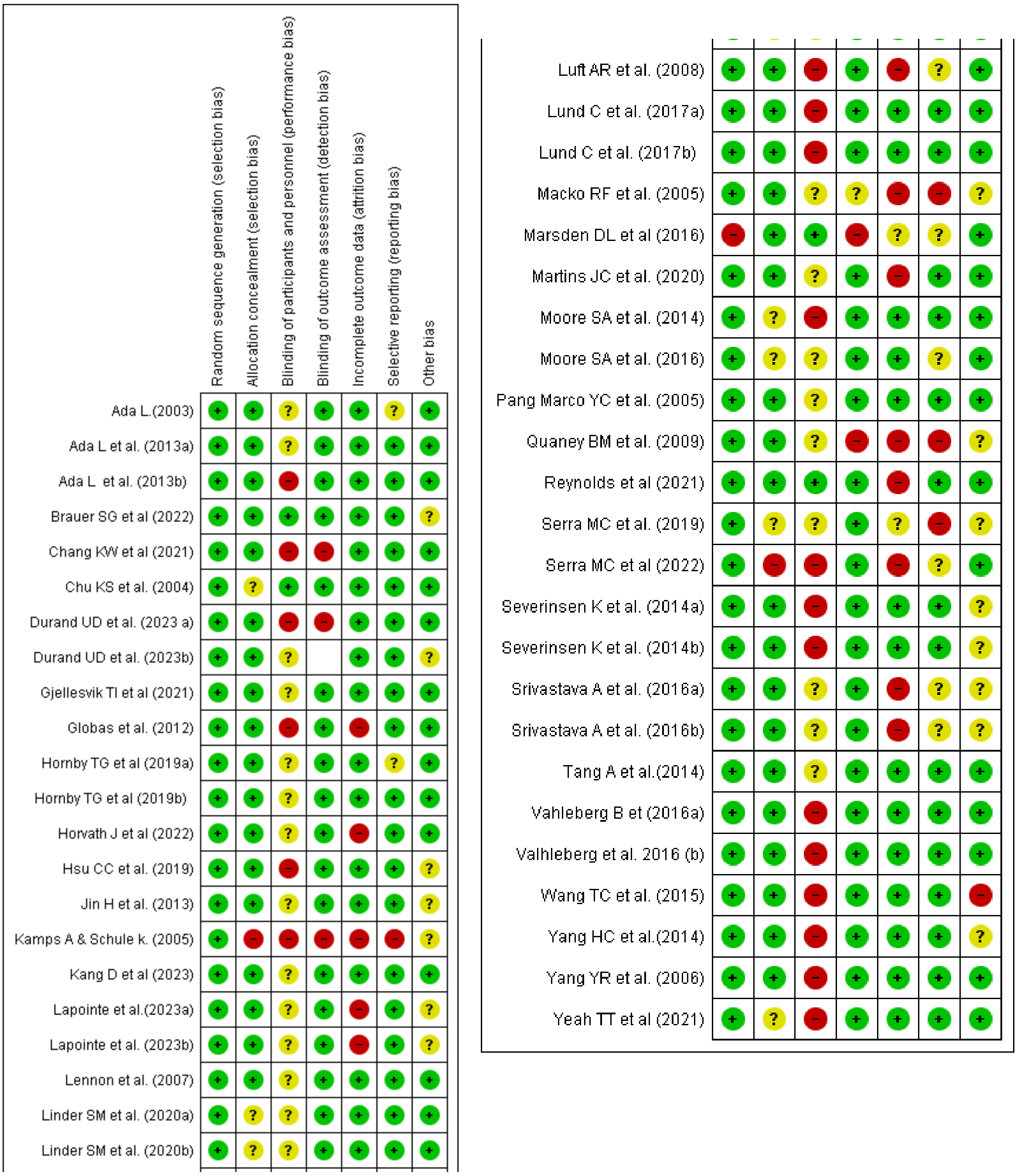

Fig. S2. Forest plot showing physical activity effect on maximal fitness (VO2) after removing low quality studies. The effect is shown by the mean difference (MD) with a random effect of each subgroup and overall effect; significance at $p < 0.05$.

	Experimental			Control				Mean Difference
**Study or Subgroup**	**Mean**	**SD**	**Total**	**Mean**	**SD**	**Total**	**Weight**	**IV, Fixed, 95% CI**
**3.1.1 Aerobic training**								
Durand UD et al. (2023 a)	32.4	11.7	13	27.8	10.7	13	0.7%	4.60 [-4.02, 13.22]
Durand UD et al. (2023b)	27.8	10.7	13	24.9	6.2	13	1.1%	2.90 [-3.82, 9.62]
Gjellesvik TI et al (2021)	29	10.3	33	27.9	7	31	2.8%	1.10 [-3.19, 5.39]
Globas et al. (2012)	24.4	6.6	18	20.9	7.8	18	2.3%	3.50 [-1.22, 8.22]
Hornby TG et al (2019a)	14	4.2	28	14	4.9	28	9.0%	0.00 [-2.39, 2.39]
Horvath J et al (2022)	11.9	8.1	19	12.45	8.4	16	1.7%	-0.55 [-6.05, 4.95]
Hsu CC et al. (2019)	13.56	8.5	15	11.94	0.65	15	2.8%	1.62 [-2.69, 5.93]
Lapointe et al.(2023a)	21.9	6.1	15	18.7	8	13	1.8%	3.20 [-2.13, 8.53]
Lapointe et al. (2023b)	24.1	4.9	13	18.7	8	13	2.0%	5.40 [0.30, 10.50]
Lennon et al. (2007)	12	2.2	24	11.1	1.9	24	38.2%	0.90 [-0.26, 2.06]
Linder SM et al. (2020a)	18.7	5.6	16	15.8	3.5	13	4.6%	2.90 [-0.44, 6.24]
Linder SM et al. (2020b)	18.7	1.9	15	15.8	3.5	13	11.4%	2.90 [0.77, 5.03]
Lund C et al. (2017a)	19.64	6.1	13	16	5.2	16	3.0%	3.64 [-0.54, 7.82]
Quaney BM et al. (2009)	15.47	5.13	19	14.39	4.99	19	5.0%	1.08 [-2.14, 4.30]
Reynolds et al (2021)	20.1	4.9	10	17.4	4.14	10	3.3%	2.70 [-1.28, 6.68]
Severinsen K et al. (2014a)	20.5	9.6	13	16.06	10	16	1.0%	4.44 [-2.72, 11.60]
Tang A et al.(2014)	17.4	7	21	16.6	5.3	25	3.9%	0.80 [-2.84, 4.44]
**Subtotal (95% CI)**			**298**			**296**	**94.6%**	**1.60 [0.86, 2.34]**
Heterogeneity: Chi² = 11.57, df = 16 (P = 0.77); I² = 0%								
Test for overall effect: Z = 4.24 (P < 0.0001)								
**3.1.3 Mixed training**								
Moore SA et al. (2014)	21	5	20	18	5	20	5.4%	3.00 [-0.10, 6.10]
**Subtotal (95% CI)**			**20**			**20**	**5.4%**	**3.00 [-0.10, 6.10]**
Heterogeneity: Not applicable								
Test for overall effect: Z = 1.90 (P = 0.06)								
**Total (95% CI)**			**318**			**316**	**100.0%**	**1.67 [0.95, 2.39]**
Heterogeneity: Chi² = 12.32, df = 17 (P = 0.78); I² = 0%								
Test for overall effect: Z = 4.56 (P < 0.00001)								
Test for subgroup differences: Chi² = 0.74, df = 1 (P = 0.39), I² = 0%								

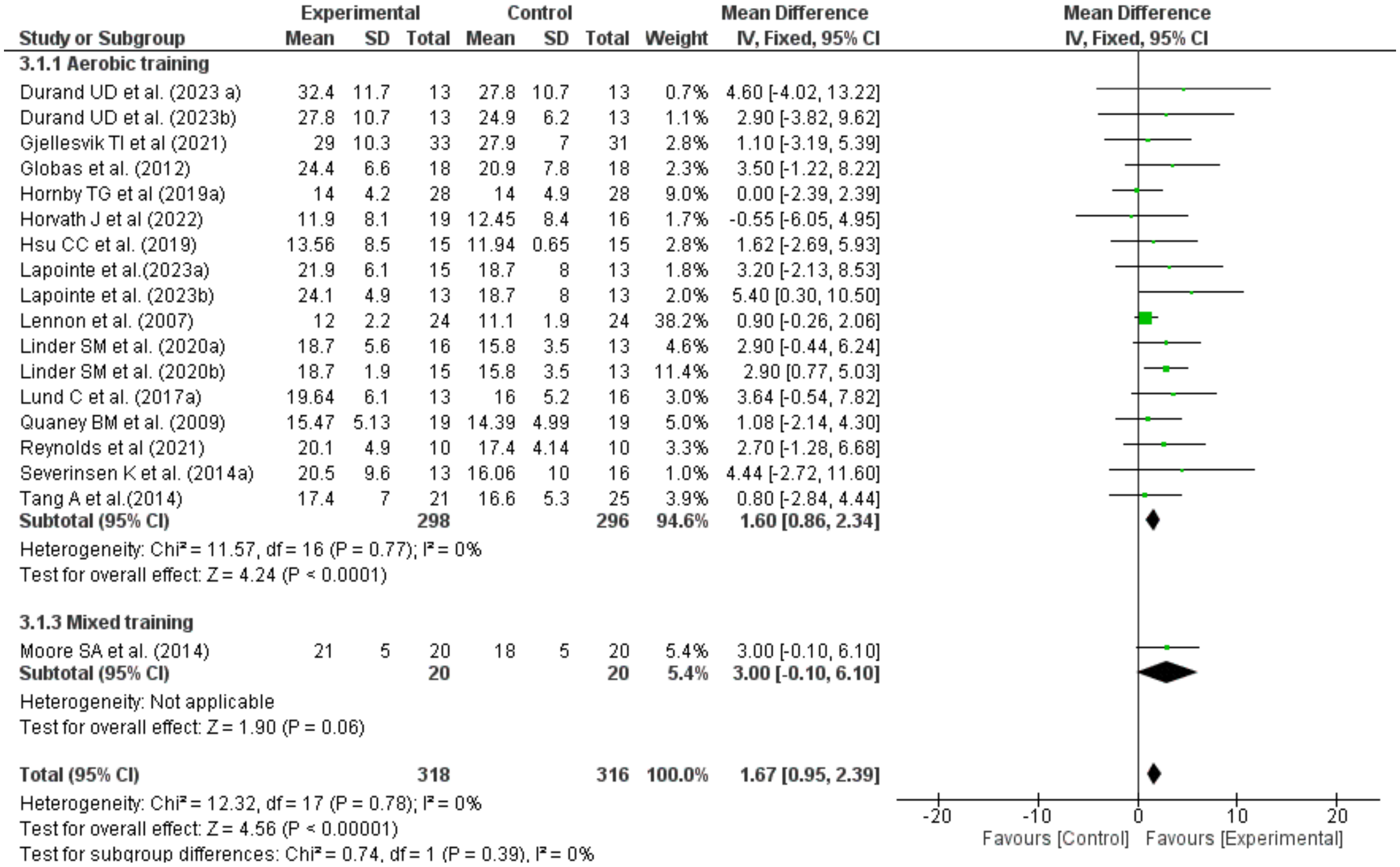

Fig. S3. Forest plot showing physical activity effect on sub-maximal fitness (6MWT) after removing low quality studies. The effect is shown by the mean difference (MD) with a random effect of each subgroup and overall effect; significance at $p < 0.05$.

	Control			Experimental				Mean Difference
Study or Subgroup	Mean [m]	SD [m]	Total	Mean [m]	SD [m]	Total	Weight	IV, Random, 95% CI
**3.2.1 Aerobic training**								
Ada L et al. (2013a)	289	131	34	263	115	34	4.6%	26.00 [-32.59, 84.59]
Ada L et al. (2013b)	274	131	34	258	116	34	4.6%	16.00 [-42.82, 74.82]
Brauer SG et al (2022)	423	116	53	370	101	55	5.1%	53.00 [11.92, 94.08]
Durand UD et al. (2023 a)	187	124.5	13	163.6	104.7	13	3.7%	23.40 [-65.03, 111.83]
Durand UD et al. (2023b)	261.1	132.9	13	163.6	104.7	13	3.6%	97.50 [5.53, 189.47]
Globas et al. (2012)	332.1	138	18	265.9	177	18	3.3%	66.20 [-37.48, 169.88]
Hornby TG et al (2019a)	210	112	28	193	108	32	4.7%	17.00 [-38.87, 72.87]
Horvath J et al (2022)	312.6	142.4	19	232	112	16	3.8%	80.60 [-3.73, 164.93]
Hsu CC et al. (2019)	380.4	47.4	15	315.6	56.3	15	5.1%	64.80 [27.56, 102.04]
Lund C et al. (2017b)	313.46	129	14	326.43	121	16	3.7%	-12.97 [-102.87, 76.93]
Severinsen K et al. (2014a)	332	115	16	350.5	138	16	3.7%	-18.50 [-106.52, 69.52]
Srivastava A et al. (2016a)	290	67.13	15	278.5	71.74	15	4.8%	11.50 [-38.22, 61.22]
Srivastava A et al. (2016a)	285.38	85.1	15	290	67.13	15	4.7%	-4.62 [-59.47, 50.23]
Tang A et al.(2014)	298.1	134.2	22	331.5	149.2	25	3.9%	-33.40 [-114.43, 47.63]
Wang TC et al. (2015)	168.4	114.8	25	156.6	117.3	25	4.4%	11.80 [-52.54, 76.14]
Yang HC et al.(2014)	284.5	139.3	15	241.4	146	15	3.3%	43.10 [-59.02, 145.22]
Yeah TT et al (2021)	168.87	107.67	18	150.14	102.45	18	4.3%	18.73 [-49.93, 87.39]
**Subtotal (95% CI)**			**367**			**375**	**71.3%**	**30.27 [15.17, 45.37]**
Heterogeneity: Tau² = 0.00; Chi² = 15.87, df = 16 (P = 0.46); I² = 0%								
Test for overall effect: Z = 3.93 (P < 0.0001)								
**3.2.2 Resistance training**								
Lund C et al. (2017b)	326.61	127	13	326.43	121	16	3.6%	0.18 [-90.82, 91.18]
Severinsen K et al. (2014b)	316.6	102	14	350.5	138	16	3.8%	-33.90 [-120.08, 52.28]
Vahleberg B et (2016a)	361.7	118	34	366.9	99.8	33	4.8%	-5.20 [-57.47, 47.07]
Valhleberg et al. 2016 (b)	420	118	20	23	385	170	4.0%	397.00 [319.39, 474.61]
Yang YR et al. (2006)	392.8	54.2	24	341.3	126.8	24	4.7%	51.50 [-3.67, 106.67]
**Subtotal (95% CI)**			**105**			**259**	**20.9%**	**81.82 [-60.02, 223.66]**
Heterogeneity: Tau² = 24766.07; Chi² = 84.41, df = 4 (P < 0.00001); I² = 95%								
Test for overall effect: Z = 1.13 (P = 0.26)								
**3.2.3 Mixed trainings**								
Martins JC et al. (2020)	334	126	18	320	124	18	3.9%	14.00 [-67.67, 95.67]
Moore SA et al. (2016)	513	131	20	441	126	20	4.0%	72.00 [-7.66, 151.66]
**Subtotal (95% CI)**			**38**			**38**	**7.9%**	**43.72 [-13.30, 100.75]**
Heterogeneity: Tau² = 0.00; Chi² = 0.99, df = 1 (P = 0.32); I² = 0%								
Test for overall effect: Z = 1.50 (P = 0.13)								
**Total (95% CI)**			**510**			**672**	**100.0%**	**39.24 [10.00, 68.48]**
Heterogeneity: Tau² = 3966.58; Chi² = 106.29, df = 23 (P < 0.00001); I² = 78%								
Test for overall effect: Z = 2.63 (P = 0.009)								
Test for subgroup differences: Chi² = 0.68, df = 2 (P = 0.71), I² = 0%								

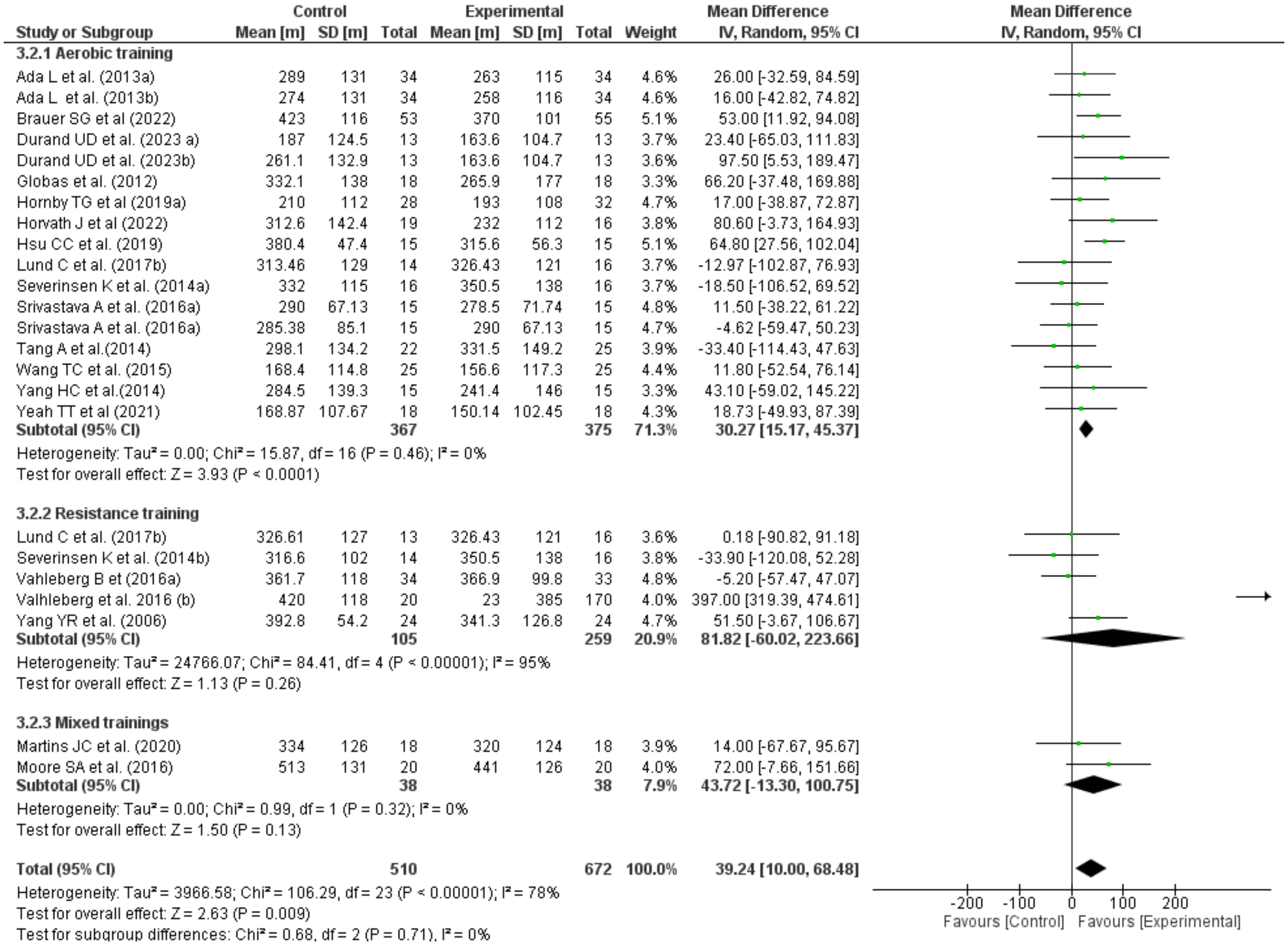

Fig. S4. Sensitivity analysis according to intensity training on sub-maximal fitness (6MWT). The effect is shown by the mean difference (MD) with a random effect of each subgroup and overall effect; significance at $p < 0.05$.

Study or Subgroup	Experimental Mean	Experimental SD	Experimental Total	Control Mean	Control SD	Control Total	Weight	Mean Difference IV, Fixed, 95% CI
**2.2.1 High intensity**								
Globas et al. (2012)	332.1	138	18	265.9	177	18	1.0%	66.20 [-37.48, 169.88]
Hornby TG et al (2019a)	210	112.5	28	193	108	32	3.4%	17.00 [-39.01, 73.01]
Jin H et al. (2013)	219.4	64.3	65	213.7	51.7	63	26.0%	5.70 [-14.48, 25.88]
Lund C et al. (2017a)	326.61	127	13	326.43	121	16	1.3%	0.18 [-90.82, 91.18]
Lund C et al. (2017b)	313.46	129	14	326.4	121	16	1.3%	-12.94 [-102.84, 76.96]
Severinsen K et al. (2014a)	332	115	13	350.5	138	16	1.2%	-18.50 [-110.59, 73.59]
Severinsen K et al. (2014b)	316.6	102	14	350.5	138	16	1.4%	-33.90 [-120.08, 52.28]
Tang A et al.(2014)	298.1	134.2	22	331.5	149.2	25	1.6%	-33.40 [-114.43, 47.63]
**Subtotal (95% CI)**			**187**			**202**	**37.2%**	**3.46 [-13.41, 20.32]**
Heterogeneity: Chi² = 3.55, df = 7 (P = 0.83); I² = 0%								
Test for overall effect: Z = 0.40 (P = 0.69)								
**2.2.2 Moderate intensity**								
Ada L et al. (2013b)	289	131	34	263	115	34	3.1%	26.00 [-32.59, 84.59]
Brauer SG et al (2022)	423	116	53	370	101	55	6.3%	53.00 [11.92, 94.08]
Hsu CC et al. (2019)	380.4	47.4	15	315.6	56.3	15	7.6%	64.80 [27.56, 102.04]
Kamps A & Schule k. (2005)	237.84	115.66	16	195.29	88.25	15	2.0%	42.55 [-29.60, 114.70]
Macko RF et al. (2005)	922	79	32	868	100	29	5.1%	54.00 [8.46, 99.54]
Marsden DL et al (2016)	494	67	10	456	101	10	1.9%	38.00 [-37.12, 113.12]
Martins JC et al. (2020)	334	126	18	320	124	18	1.6%	14.00 [-67.67, 95.67]
Pang Marco YC et al. (2005)	392.7	151.1	32	342.4	133.4	31	2.1%	50.30 [-20.03, 120.63]
Serra MC et al (2022)	343.85	37.66	19	268.98	36.38	20	19.6%	74.87 [51.61, 98.13]
Yang YR et al. (2006)	392.8	54.2	24	341.3	126.8	24	3.5%	51.50 [-3.67, 106.67]
**Subtotal (95% CI)**			**253**			**251**	**52.8%**	**59.02 [44.85, 73.18]**
Heterogeneity: Chi² = 5.02, df = 9 (P = 0.83); I² = 0%								
Test for overall effect: Z = 8.17 (P < 0.00001)								
**2.2.3 Low intensity**								
Chang KW et al (2021)	255.1	166.1	8	249.7	113.8	8	0.5%	5.40 [-134.12, 144.92]
Durand UD et al. (2023 a)	187	124.5	13	163.6	104.7	13	1.4%	23.40 [-65.03, 111.83]
Durand UD et al. (2023b)	261.1	132.9	13	163.6	104	13	1.3%	97.50 [5.77, 189.23]
Srivastava A et al. (2016a)	290	67.13	15	278.5	71.7	15	4.3%	11.50 [-38.21, 61.21]
Wang TC et al. (2015)	168.4	114.8	25	156.6	117.3	26	2.6%	11.80 [-51.90, 75.50]
**Subtotal (95% CI)**			**74**			**75**	**10.0%**	**23.62 [-8.84, 56.07]**
Heterogeneity: Chi² = 2.92, df = 4 (P = 0.57); I² = 0%								
Test for overall effect: Z = 1.43 (P = 0.15)								
**Total (95% CI)**			**514**			**528**	**100.0%**	**34.79 [24.50, 45.08]**
Heterogeneity: Chi² = 36.45, df = 22 (P = 0.03); I² = 40%								
Test for overall effect: Z = 6.63 (P < 0.00001)								
Test for subgroup differences: Chi² = 24.96, df = 2 (P < 0.00001), I² = 92.0%								

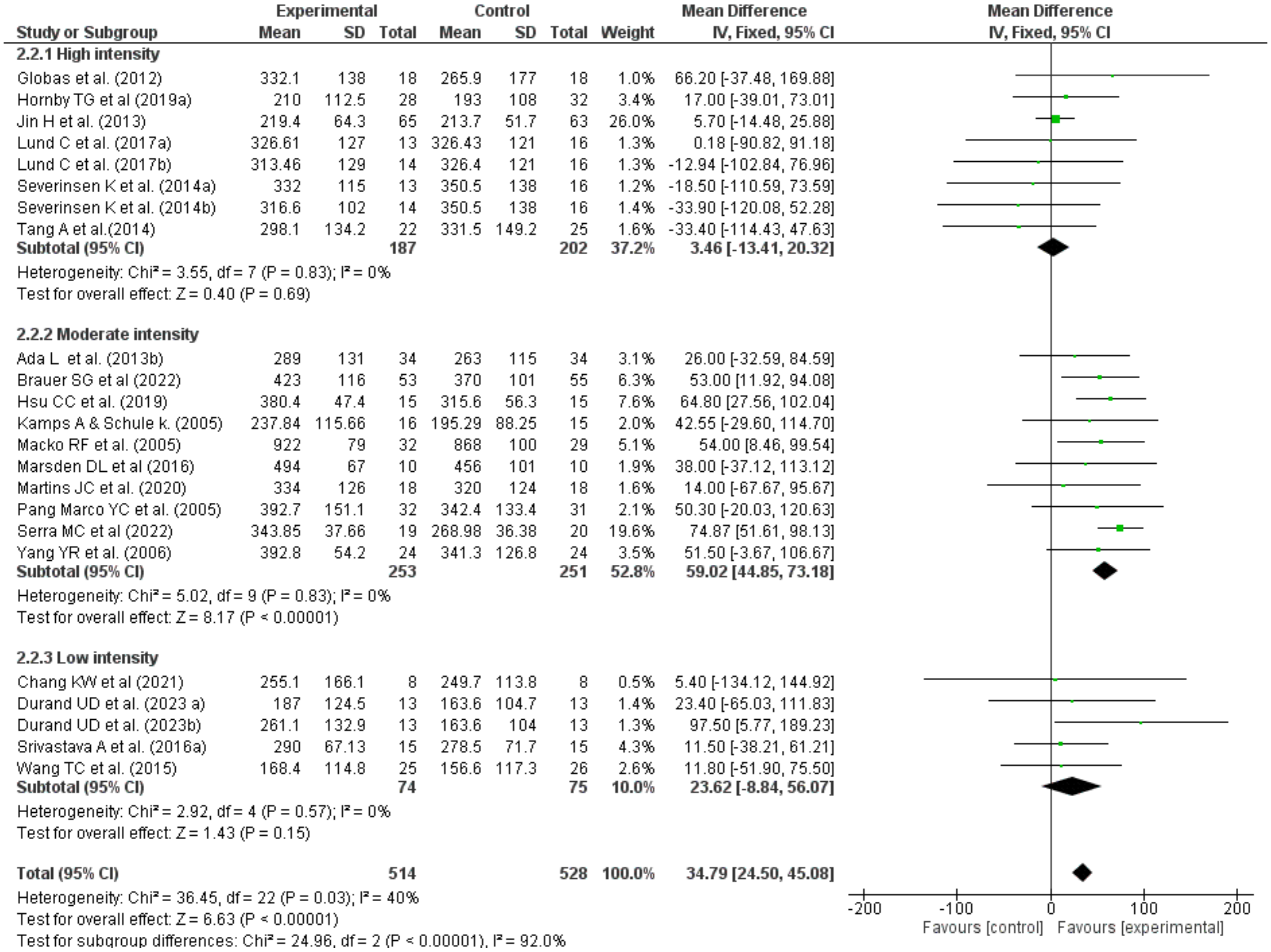

Supplement: Supplementary file 1 [file JRM-57-43359-s1.pdf]
