# Supplementary material for: OPTIMIZING PHYSICAL FITNESS IN CHRONIC STROKE PATIENTS: THE IMPACT OF EXERCISE TRAINING MODALITY AND DOSAGE ON MAXIMAL AND SUB-MAXIMAL FITNESS – A SYSTEMATIC REVIEW AND META-ANALYSIS
Source: J Rehabil Med. 2025 Aug 11;57:43359. doi: 10.2340/jrm.v57.43359 (PMC12359817; doi:10.2340/jrm.v57.43359)
Supplement: Supplementary file 2 [file JRM-57-43359-s2.pdf]

Appendix 1: Search strategies

Pubmed Search strategies

**#1**	stroke[MeSH Terms]
**#2**	"chronic stroke"[Title/Abstract] OR "cerebrovascular accident"[Title/Abstract] OR “cerebrovascular accidents”[Title/Abstract] OR “brain vascular accident”[Title/Abstract] OR “brain vascular accidents"[Title/Abstract]
**#3**	#1 OR #2
**#4**	walking[MeSH Terms]
**#5**	walking[Title/Abstract] OR walk*[Title/Abstract] OR Gait[Title/Abstract] OR "gait training"[Title/Abstract]
**#6**	#4 OR #5
**#7**	exercise[MeSH Terms]
**#8**	exercise[Title/Abstract] OR exercises[Title/Abstract] OR "physical activity"[Title/Abstract] OR "physical activities"[Title/Abstract] OR "aerobic exercise"[Title/Abstract] OR "exercise training"[Title/Abstract
**#9**	#7 OR #8
**#10**	recovery of function"[MeSH Terms]
**#11**	(function[Title/Abstract] OR function recover*[Title/Abstract] OR activity[Title/Abstract] OR "activity limitation"[Title/Abstract] OR "physical fitness"[Title/Abstract] OR "quality of life"[Title/Abstract] OR "Community Participation"[Title/Abstract] OR participation[Title/Abstract]
**#12**	#10 OR #11
**#13**	#3 AND #6 AND #9 AND #12

MeSH: Medical Subject Heading.

Cochrane library search strategy

ID	Search Hits
#1	(stroke OR "chronic stroke" OR "cerebrovascular accident" OR "cerebrovascular accidents" OR "brain vascular accident" OR "brain vascular accidents"):ti,ab,kw (Word variations have been searched)
#2	MeSH descriptor: [Stroke] explode all trees
#3	walking OR walk* OR gait OR "gait training"
#4	MeSH descriptor: [Walking] explode all trees
#5	exercise OR exercises OR "physical activity" OR "physical activities" OR "aerobic exercise" OR "exercise training"
#6	MeSH descriptor: [Exercise] explode all trees
#8	"recovery of function" OR function OR function AND recover* OR activity OR "activity limitation" OR "physical fitness" OR "quality of life" OR "Community Participation"
#9	MeSH descriptor: [International Classification of Functioning, Disability and Health] explode all trees
#10	#7 OR #8
#11	#5 OR #6
#12	#3 OR #4
#13	#1 OR #2
#14	#10 AND #11
#15	#12 AND #13
#16	#14 AND #15

Scopus search strategy: (TITLE-ABS-KEY (stroke OR "chronic stroke" OR "cerebrovascular accident" OR "cerebrovascular accidents" OR "brain vascular accident" OR "brain vascular accidents") AND TITLE-ABS-KEY (walking OR walk* OR gait OR "gait training") AND TITLE-ABS-KEY (exercise OR exercises OR "physical activity" OR "physical activities" OR "aerobic exercise" OR "exercise training") AND TITLE-ABS-KEY ("recovery of function" OR function OR function AND recover* OR activity OR "activity limitation" OR "physical fitness" OR "quality of life" OR "Community Participation" OR participation)) AND PUBYEAR > 2007 AND PUBYEAR < 2019

Sciences direct: stroke, stroke rehabilitation, walking, exercise, physical activity
